# Prognosis of Patients with Chronic Hepatitis C Genotype 1b Infection Treated Using Daclatasvir/Asunaprevir after Sustained Virologic Response: A 6-Year Multicenter Prospective Observational Study

**DOI:** 10.3390/medicina59081436

**Published:** 2023-08-08

**Authors:** Jae-Hyun Yoon, Sung-Eun Kim, Su-Hyeon Cho, Gi-Ae Kim, Yewan Park, Ji-Won Park, Seong-Hee Kang, Young-Sun Lee, Jeong-Han Kim

**Affiliations:** 1 Department of Internal Medicine, School of Medicine, Chonnam National University Hospital, Gwangju 61469, Republic of Korea; zenmake14@gmail.com (J.-H.Y.); hyekyo0122@naver.com (S.-H.C.); 2 Department of Internal Medicine, College of Medicine, Hallym University, Anyang 14068, Republic of Korea; sekim@hallym.or.kr (S.-E.K.); miunorijw@hallym.or.kr (J.-W.P.); 3 Department of Internal Medicine, School of Medicine, Kyung Hee University, Seoul 02447, Republic of Korea; antiankle@hanmail.net (G.-A.K.); yewanish@gmail.com (Y.P.); 4 Department of Internal Medicine, Inje University Sanggye Paik Hospital, Seoul 01757, Republic of Korea; shkang0114@gmail.com; 5 Department of Internal Medicine, Korea University Medical Center, Seoul 08308, Republic of Korea; lys810@korea.ac.kr; 6 Department of Internal Medicine, School of Medicine, Konkuk University, Seoul 05030, Republic of Korea; 7 Research Institute of Medical Science, School of Medicine, Konkuk University, Seoul 05030, Republic of Korea

**Keywords:** hepatitis C, antiviral agents, sustained virologic response, hepatocellular carcinoma, liver cirrhosis

## Abstract

*Aim and Objectives*: Direct-acting antiviral (DAA) therapy can cure chronic hepatitis C (CHC), and daclatasvir (DCV)/asunaprevir (ASV) was the first interferon-free DAA therapy introduced in Korea. Patients who achieve sustained virologic response (SVR) after DAA treatment are expected to have good prognoses. Therefore, in this study, we aimed to investigate the prognosis of these patients. *Materials and Methods*: This multicenter prospective observational study included patients with CHC who achieved SVR after DCV/ASV treatment. The primary endpoint was hepatocellular carcinoma (HCC) occurrence, which was reviewed annually. *Results*: We included 302 patients (median follow-up duration: 38 [16.5–60.0] months; median age: 58 [49–67] years) in the study. Cirrhosis was observed in 103 patients (34.1%), and the median Child–Pugh score was 5.0. HCC occurred in 16 patients (5.3%) within six years post-SVR; these patients were older and had higher cirrhosis prevalence, alpha-fetoprotein levels, and fibrosis-4 index scores than did those without HCC development. Cox proportional hazards analysis revealed that age > 71 years (*p* = 0.005) and cirrhosis (*p* = 0.035) were significant risk factors for HCC occurrence. *Conclusions*: Although the prognoses of patients who achieved SVR with DCV/ASV therapy were generally good, the risk for HCC was present, especially in older patients and in those with cirrhosis. Hence, early treatment at younger ages and regular follow-up surveillance after achieving SVR are warranted.

## 1. Introduction

Hepatitis C virus (HCV) infection results in a substantial medical and economic burden globally; in 2019, 58 million people worldwide were estimated to have a chronic HCV (CHC) infection [1]. Although CHC infections are often asymptomatic, they may progress to cirrhosis over 20–30 years. The annual incidence rates of hepatocellular carcinoma (HCC) and hepatic decompensation are 1–7% and 3–6%, respectively, among individuals with CHC [2]. In South Korea, HCV infection is the second most common cause of chronic liver diseases [3]. 

From a therapeutic point of view, the development of direct-acting antivirals (DAAs) has been a turning point in CHC treatment. DAAs have a high efficacy for viral eradication and an excellent safety profile; hence, they have replaced interferon-based treatments as the first-line therapy for HCV. In particular, combination therapy with daclatasvir (DCV) and asunaprevir (ASV), the first interferon-free regimen, was developed to overcome the shortcomings of interferon therapy and has replaced interferon-based therapy in clinical practice [4,5,6]. The primary goal of therapy in patients with CHC is to achieve a sustained virologic response (SVR). 

Patients with CHC that achieve an SVR have demonstrated decreased rates of life-threatening complications, all-cause mortality, decompensated cirrhosis, and HCC [7,8]. In particular, SVR achievement in patients with CHC predicts HCC-free survival with both interferon-based regimens and DAA therapy, along with fibrosis regression [9,10]. SVR achievement in CHC treatment is an important factor in determining the prognosis of chronic liver disease, with outcomes such as a decreased rate of HCC development or regression of hepatic cirrhosis or fibrosis. However, the actual incidence of HCC after DAA-induced HCV eradication in patients with and without cirrhosis, and according to the fibrosis stage, is unclear [11]. Obtaining accurate estimates of HCC incidence after DAA-induced eradication is critical because HCC incidence is positively correlated with the potential benefits of screening and is the main determinant of the cost-effectiveness of HCC screening after HCV eradication [12]. However, the long-term incidence rate of HCC and the risk of developing HCC after achieving SVR have not been fully described for each DAA.

Recently, Fujii et al. demonstrated that DSV/ASV combination treatment was a critical factor for the surveillance of HCC development in Japan [13]. However, their study had some limitations because it had a high dropout rate and patients who had recently received treatment for HCC before the administration of DSV/ASV therapy were enrolled. Although several studies have reported clinical outcomes after DSV/ASV treatment [13,14,15], the long-term clinical outcomes of developing HCC and recurrence/reinfection after achieving SVR have not been fully described in patients who underwent DCV/ASV therapy. Therefore, this study aimed to investigate the long-term outcomes, including HCC development, risk factors of HCC, regression of hepatic fibrosis, and HCV recurrence or reinfection of DCV/ASV treatment in patients with CHC who achieved SVR.

## 2. Materials and Methods

### 2.1. Patients

This prospective observational multicenter study included six teaching hospitals in South Korea and was approved by the ethics committees of each hospital. Informed consent was obtained from all participants. Patients with CHC who achieved SVR after DCV/ASV treatment were enrolled between March 2016 and February 2021; the final follow-up month was August 2022. The inclusion criteria were as follows: (1) the presence of CHC with the achievement of SVR after DCV/ASV treatment and (2) age ≥ 18 years. The exclusion criteria were as follows: (1) unwillingness to be enrolled in the study; (2) inability to understand or provide informed consent; (3) presence of malignant tumors, including HCC; (4) coinfection with chronic hepatitis B or human immunodeficiency virus; (5) a history of liver transplantation; and (6) a history of CHC treatment. Patients with compensated cirrhosis were included in the study.

SVR was defined as undetectable HCV-RNA levels 12 weeks after treatment completion. Cirrhosis was defined based on (1) histologic confirmation, (2) the presence of cirrhotic configuration of the liver and/or splenomegaly revealed by radiologic examinations such as abdominal ultrasonography or computed tomography, (3) esophageal-gastric varices detected using upper endoscopy or cross-sectional images, and (4) the presence of thrombocytopenia [16]. DCV capsules (60 mg once daily) and ASV tablets (100 mg twice daily) were administered orally for 24 weeks, according to the manufacturer’s specifications. The aspartate aminotransferase (AST) to platelet ratio index (APRI) and fibrosis-4 index (FIB-4) were measured in the enrolled patients to evaluate the degree of hepatic fibrosis. These markers were calculated using the following formulae: APRI = ([AST level/AST upper limit of normal]/platelet count [10^9^/L]) × 100; FIB-4 index = age (years) × AST (IU/L)/(platelet count (10^9^/L) × (alanine aminotransferase [IU/L]^1/2^) [17,18]. APRI and FIB-4 were evaluated at baseline and annually.

The clinical outcomes were as follows: (1) HCC occurrence as the primary endpoint and (2) HCV recurrence or reinfection as the secondary endpoint. Hepatic decompensation was defined as overt ascites, hepatic encephalopathy, variceal bleeding, bacterial infection, or deterioration of liver dysfunction, defined as serum bilirubin levels ≥ 3 mg/dL. During the follow-up period, all enrolled patients underwent liver function tests, tests for HCV-RNA and alpha-fetoprotein (AFP) levels, and radiologic examinations, such as abdominal ultrasonography or computed tomography, at least once annually.

### 2.2. Statistical Analysis 

Continuous variables are expressed as median (interquartile range), while categorical variables are presented as numbers (%). The Mann–Whitney U, chi-squared, and Fisher’s exact tests were conducted to compare variables between groups, as appropriate. The Wilcoxon signed-rank test was used to assess significant changes in liver function and noninvasive fibrosis scores at two different time points. Cumulative HCC occurrence was evaluated using the Kaplan–Meier method, and differences were determined using the log-rank test. 

Univariate and multivariate analyses were performed to identify independent predictors of HCC occurrence using a Cox regression hazard model (forward). Significant factors that predicted HCC development after treatment in patients with CHC who achieved SVR were extracted. Continuous variables were categorized according to the area under the receiver operating characteristic (ROC) curve to predict HCC development. New cutoff values were defined using the Youden index set to the maximum level and generated automatically using MedCalc^®^ software. Statistical significance was set at *p* < 0.05, and all analyses were performed using SPSS (version 27.0; IBM Corp., Armonk, NY, USA) and MedCalc version 22.005 (MedCalc Software, Mariakerke, Belgium).

## 3. Results

### 3.1. Baseline Characteristics

This study enrolled 302 patients, including 148 (49.0%) males, and the median age was 58 years (Table 1). The median follow-up duration was 38 months, and 103 patients (34.1%) had cirrhosis. Sixty (19.9%) patients had a significant history of alcohol consumption. The median AFP and pre-treatment HCV-RNA levels were 3.8 ng/mL and 905,000 IU/mL, respectively, and the median Child–Pugh and model for end-stage liver disease (MELD) scores were 5.0 and 7.0, respectively. The median pre-treatment APRI and FIB-4 scores were 0.6 and 2.4, respectively, and the median baseline APRI and FIB-4 scores were 0.4 and 2.1, respectively.

### 3.2. Prognosis after Achieving SVR

During the follow-up period, HCC occurred in 16 patients: in 5 (1.7%), 7 (2.3%), 9 (3.0%), 12 (4.0%), 13 (4.3%), and 16 (5.3%) patients within 1, 2, 3, 4, 5, and 6 years, respectively (Table 2). Recurrence or reinfection occurred in two 69-year-old females (0.7%); both patients had cirrhosis with baseline HCV-RNA levels of 3,450,000 and 6,600,000 IU/mL, respectively. The patients’ HCV-RNA levels were detectable at three and eight months after achieving SVR, respectively (Table 3).

Decompensation events occurred in 8 patients: 2 (0.7%), 4 (1.3%), 7 (2.3%), 7 (2.3%), 7 (2.3%), and 8 (2.6%) patients within 1, 2, 3, 4, 5, and 6 years, respectively. These patients either had cirrhosis or were older (69 years vs. 57 years, *p* = 0.011) than the rest of the patients. Furthermore, these patients had higher MELD (9 vs. 7, *p* = 0.008), APRI (0.6 vs. 0.4, *p* = 0.032), and FIB-4 (3.9 vs. 2.1, *p* = 0.005) scores than the rest of the patients.

### 3.3. Changes in Liver Function

The Child–Pugh and MELD scores were compared annually with the baseline scores (Table 4). The Child–Pugh score did not significantly change during the follow-up period. In contrast, the MELD score gradually decreased from the second year onwards.

### 3.4. Changes in Noninvasive Fibrosis Score

The APRI and FIB-4 scores were compared annually with the pre-treatment scores (Table 5); both APRI and FIB-4 scores significantly decreased (*p* < 0.005).

### 3.5. Risk Factors for HCC Occurrence

Sixteen patients developed HCC during the follow-up period. These patients were older (74 years vs. 57 years, *p* < 0.001) and had a higher cirrhosis incidence (68.8% vs. 32.2%, *p* = 0.005), higher AFP levels (7.1 ng/mL vs. 3.6 ng/mL, *p* = 0.006), and higher pre-FIB-4 (3.8 vs. 2.3, *p* = 0.008), APRI (0.6 vs. 0.4, *p* = 0.034), and FIB-4 (3.8 vs. 2.1, *p* = 0.002) scores (Table 1) than did the rest of the patients.

**Figure 1:**
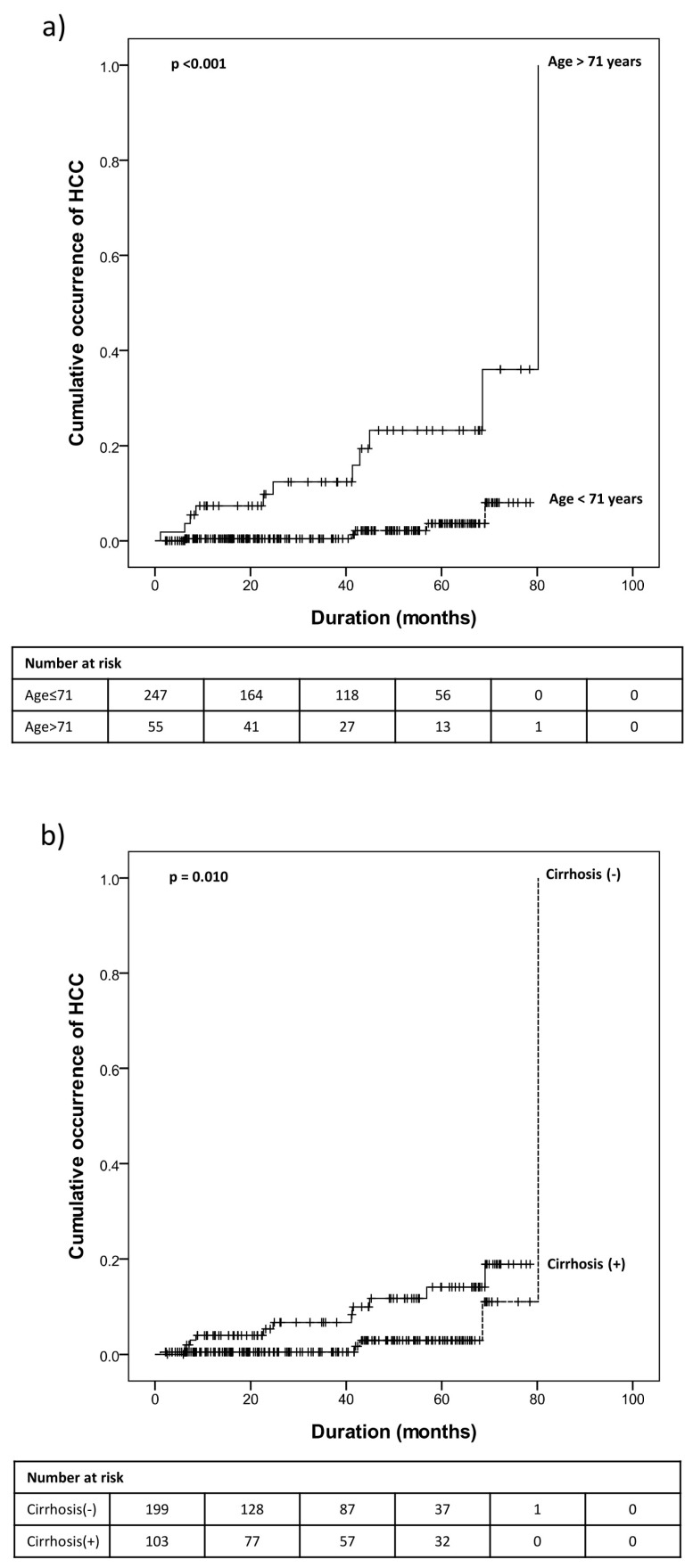
Cumulative occurrence of HCC after SVR according to (**a**) age > 71 years or not and (**b**) presence or absence of cirrhosis. HCC, hepatocellular carcinoma; SVR, sustained virologic response.

According to the area under the ROC curve and Youden index (J) analysis, cutoff levels for risk factors were as follows: (1) age, 71 years; (2) AFP, 3.85 ng/mL; (3) pre-FIB-4, 2.227; (4) APRI, 0.556; and (5) FIB-4, 2.123. These variables were all significant (*p* < 0.1) in the univariate analysis. Additionally, age and cirrhosis were significant variables in the multivariate analysis (Table 6, Figure 1). After excluding patients with cirrhosis, only age (cutoff value, 71 years) was found to be a significant factor (Table 6).

## 4. Discussion

The introduction of DAAs, which have excellent treatment efficacy and a fair safety profile, has made HCV eradication possible. Several previous studies have reported high SVR rates with various DAAs in diverse clinical settings and in many countries [19]. The achievement of SVR is associated with the prevention of liver cirrhosis progression, HCC development, and a reduction in overall mortality [7,8]. Several previous studies have demonstrated these preventive effects of DAA, and the World Health Organization has proposed a goal of worldwide HCV elimination by 2030 using DAAs [1,19].

Combination therapy with DCV/ASV, which are a nonstructural protein 5A inhibitor and nonstructural protein 3/4A protease inhibitor, respectively, was introduced in Korea in August 2015 as one of the earliest interferon-free DAA regimens for treating HCV genotype 1b infection. Real-world data from a multicenter study conducted in South Korea demonstrated an SVR of 91.6% among 278 patients treated with DCV/ASV [20].

The present study evaluated 302 patients who achieved SVR with DCV/ASV and showed an extremely low rate of HCV recurrence or reinfection (0.7%, 2 of 302 patients) during 38 months of follow-up after achieving SVR. The patients demonstrated improved reserve liver function after achieving SVR. However, 5.3% of the enrolled patients developed HCC. Among the factors associated with HCC development, age > 71 years and the presence of cirrhosis were observed to be closely related in the multivariate analysis. If patients with cirrhosis were excluded, age > 71 years was the only risk factor.

There have been no previous reports regarding the long-term recurrence or reinfection rates after HCV elimination with DCV/ASV. To the best of our knowledge, the present study is the first to report long-term follow-up results of HCV recurrence or reinfection rates after SVR achievement with DCV/ASV. Furthermore, this study demonstrated a persistent virologic response without recurrence or reinfection for more than three years in most patients (99.3%). Two women, each aged 69 years, who underwent 24 weeks of DCV/ASV therapy for HCV genotype 1b infection experienced HCV recurrence or reinfection at three and eight months, respectively, after achieving SVR. Both patients had underlying compensated cirrhosis and high HCV-RNA levels. The presence of cirrhosis and a high HCV-RNA viral load have been proposed as risk factors for DAA treatment failure. 

Previous studies have reported a reversal effect on liver function impairment and fibrosis after hepatitis C elimination [21,22]. Similarly, the present study also showed a decreasing MELD score, which represents reserve liver function, after three years of DCV/ASV therapy; this trend continued for up to six years after therapy. In addition, APRI and FIB-4 scores indicating the fibrotic burden of the liver exhibited improvement after achieving SVR (baseline), compared with the pre-treatment score, and persisted throughout the entire observation period.

There has been ongoing successful HCV eradication with the introduction of DAAs, and the number of patients who have achieved SVR is also increasing. However, eventual HCC development, which is difficult to avoid, is the main burden in patients with successful SVR, especially in those with high-risk features [23]. In the present study, 16 (5.3%) of 302 patients experienced HCC development during a median follow-up duration of 38 months after achieving SVR. Of these patients, 31.2% were non-cirrhotic, and most patients diagnosed with HCC were male (11 patients, 68.6%). Patients’ age at HCC diagnosis ranged from 50 to 90 years, and the serum AFP level showed a diverse range (2.8–138.6 ng/mL). On multivariate analysis, age > 71 years and liver cirrhosis were associated with HCC development after achieving SVR. Previous studies have reported liver cirrhosis and old age as risk factors for failure to achieve SVR [24,25]. In light of the advent of highly efficacious treatment with DAAs, HCC surveillance after achieving SVR is paramount; therefore, identifying relevant risk factors for HCC development is essential. The recommended HCC screening method in high-risk patients is ultrasonography with or without AFP levels [26,27,28,29]. Professional liver societies differ in their HCC screening recommendations after an HCV cure [11]. The American Association for the Study of Liver Diseases recommends HCC screening only in patients with pre-treatment cirrhosis [27]. The European Association for the Study of the Liver recommends HCC screening in patients with pre-treatment stage 3 (F3) fibrosis and cirrhosis [26]. The Asian Pacific Association for the Study of the Liver (APASL) recommends HCC screening in all patients after SVR, irrespective of fibrosis stage and the presence of cirrhosis [28]. Considering current results, at least older patients or patients with cirrhosis still need regular HCC screening according to current guidelines.

The present study had several limitations. First, the total number of patients diagnosed with HCC during follow-up after SVR achievement was limited. Despite enrolling a sizeable number of patients from multiple institutions and following up for more than three years, HCC developed in only 5.3% of the enrolled patients. Hence, further studies with a larger number of patients and a longer observation period than those of the present study may be necessary to investigate the risk factors for HCC development. Second, the present study included patients with an HCV genotype 1b infection treated with DCV/ASV. Therefore, this study’s results may not apply to patients with an HCV infection of other genotypes and those who received other DAAs. Third, our study has limited data about potential confounding factors such as comorbidities, medication use, and environmental factors that may influence the association between DCV/ASV treatment and prognosis. To overcome this limitation, larger data analyses are warranted. Finally, many guidelines recommend pan-genotypic agents with simplified treatment algorithms after the introduction of pan-genotypic DAA agents, such as sofosbuvir with velpatasvir or glecaprevir with pibrentasvir [30,31]. Although pan-genotypic agents may guarantee better treatment outcomes and adherence, DCV/ASV may have a cost-benefit advantage in circumstances with insufficient medical resources. 

The current data lacked information about genetic and environmental factors related to the prognosis of such patients and HCC screening strategy treatment modalities. Therefore, future research should include these factors.

## 5. Conclusions

In conclusion, patients who underwent 24 weeks of DCV/ASV treatment demonstrated a very low HCV recurrence/reinfection rate, improved liver function, and improved fibrotic burden after achieving SVR. However, patients with risk factors for HCC, such as old age (>71 years) and cirrhosis, should undergo close surveillance for HCC development. Further studies are warranted to determine the efficacy of post-SVR treatment and the factors associated with HCC development.

## Figures and Tables

**Table 1:** Patient characteristics.

	Total (n = 302)	HCC Occurrence (−) (n = 286)	HCC Occurrence (+) (n = 16)	*p*-Value	HCC Occurrence (−), in Patients without Cirrhosis (n = 194)	HCC Occurrence (+), in Patients without Cirrhosis (n = 5)	*p*-Value
Male sex	148 (49.0%)	137 (47.9%)	11 (68.6%)	0.127	96 (49.5%)	4 (80.0%)	0.369
Age (years)	58 (49–67)	57 (49–67)	74 (65–75)	<0.001 **	54 (47–63)	75 (72–76)	0.009 **
Follow-up duration (months)	38.0 (16.5–60.0)	36.9 (16.1–58.1)	41.2 (8.3–46.4)	0.603	31.4 (15.2–54.2)	70.2 (67.5–77.4)	0.603
AFP (ng/mL)	3.8 (2.6–6.6)	3.6 (2.5–6.4)	7.1 (4.5–13.4)	0.006 ***	3.1 (2.1–5.0)	5.4 (4.2–9.3)	0.175
Pre-treatment HCV RNA (IU/mL)	905,000 (188,750–2,500,000)	905,000 (185,500–2,382,500)	923,000 (604,500–4,235,500)	0.513	1,060,000 (186,000–2,682,500)	4,104,000 (1,470,000–4,498,500)	0.199
Cirrhosis	103 (34.1%)	93 (32.4%)	11 (68.8%)	0.005 **			
Significant alcohol consumption	60 (19.9%)	58 (20.2%)	2 (12.5%)	0.747	40 (20.6%)	1 (20.0%)	1.000
Child–Pugh score	5.0 (5.0–5.0)	5.0 (5.0–5.0)	5.0 (5.0–5.0)	0.316	5.0 (5.0–5.0)	5.0 (5.0–5.0)	0.666
5	282 (93.4%)	269 (93.7%)	14 (87.5%)	0.336	187 (96.4%)	5 (100.0%)	0.911
6	14 (4.6%)	13 (4.5%)	1 (6.3%)		6 (3.1%)		
7	2 (0.7%)	2 (0.7%)	0 (0%)				
8	4 (1.3%)	3 (1.0%)	1 (6.3%)		1 (0.5%)		
MELD score	7.0 (6.4–8.0)	7.0 (6.0–8.0)	7.0 (7.0–9.0)	0.179	7.0 (6.4–7.0)	6.4 (6.2–6.6)	0.214
Pre-APRI	0.6 (0.3–1.2)	0.6 (0.3–1.2)	0.8 (0.6–1.2)	0.137	0.5 (0.3–0.8)	0.8 (0.6–1.2)	0.137
Pre-FIB-4	2.4 (1.5–4.5)	2.3 (1.5–4.5)	3.8 (3.0–5.2)	0.008 **	2.0 (1.3–3.0)	3.2 (2.2–3.8)	0.089
APRI	0.4 (0.3–0.7)	0.4 (0.3–0.7)	0.6 (0.5–1.1)	0.034	0.4 (0.3–0.5)	0.4 (0.3–0.5)	0.939
FIB-4	2.1 (1.4–3.7)	2.1 (1.4–3.5)	3.8 (2.6–4.2)	0.002 **	1.7 (1.2–2.4)	2.8 (2.2–3.0)	0.035 *

Data are presented as n (%) or median (interquartile range) as appropriate. The Mann–Whitney U, chi-squared, or Fisher’s exact tests were used for comparisons as appropriate. Statistical significance is indicated by * *p* < 0.05, ** *p* < 0.01, and *** *p* < 0.001. AFP, alpha-fetoprotein; APRI, AST to platelet ratio index; AST, aspartate aminotransferase; FIB-4, fibrosis-4 index; HCV, hepatitis C virus; HCC, hepatocellular carcinoma; MELD, model for end-stage liver disease.

**Table 2:** Characteristics of patients in whom HCC occurred.

Patient	Sex	Age (Years)	Cirrhosis	Alcohol Consumption	Time after Achieving SVR (Months)	Serum AFP (ng/mL)	Child–Pugh Score	MELD Score	APRI	FIB-4
1	Male	77	+	−	45	12.92	5	9	0.638	5.072
2	Male	53	−	+	42	21.01	5	6	1.579	3.149
3	Male	75	+	−	23	13.36	5	9	0.619	3.962
4	Male	90	+	−	7	2.8	8	10	1.976	10.267
5	Male	52	+	−	69	18.31	5	7	0.418	1.883
6	Female	50	+	−	41	7.07	5	7	0.230	1.536
7	Male	80	−	−	1	7.1	5	7	0.336	2.775
8	Male	73	+	−	6	6.04	5	8	0.647	5.244
9	Female	77	+	−	9	138.6	6	9	2.290	15.036
10	Male	72	−	−	69	2.99	5	6	0.291	2.238
11	Female	75	−	−	80	5.39	5	7	0.582	4.007
12	Female	67	+	−	57	3.87	5	7	1.071	4.924
13	Male	76	−	−	43	7.2	5	7	0.370	1.957
14	Male	60	+	+	6	4.54	5	7	1.147	2.674
15	Male	74	+	−	41	7.0	5	7	0.580	3.746
16	Female	74	+	−	25	7.45	5	10	0.645	3.894

AFP, alpha-fetoprotein; APRI, AST to platelet ratio index; AST, aspartate aminotransferase; FIB-4, fibrosis-4 index; MELD, model for end-stage liver disease; SVR, sustained virologic response.

**Table 3:** Characteristics of patients with HCV recurrence or reinfection.

Patient	Sex	Age (Years)	Cirrhosis	Alcohol Consumption	Time after Achieving SVR (Months)	HCV RNA (IU/mL)	Child–Pugh Score	MELD Score	APRI	FIB-4
1	Female	69	−	−	8	6,600,000	5	6	0.321	2.087
2	Female	69	+	−	3	3,450,000	5	8	1.678	10.354

APRI, AST to platelet ratio index; AST, aspartate aminotransferase; FIB-4, fibrosis-4 index; HCV, hepatitis C virus; MELD, model for end-stage liver disease; SVR, sustained virologic response.

**Table 4:** Changes in liver function.

	Child–Pugh Score	*p*-Value (vs. Baseline)	MELD Score	*p*-Value (vs. Baseline)
Baseline	5.0 (5.0–5.0)		7.0 (6.4–8.0)	
One year later	5.0 (5.0–5.0)	0.279	7.0 (6.9–8.0)	0.827
Two years later	5.0 (5.0–5.0)	0.538	7.0 (6.4–7.9)	0.065
Three years later	5.0 (5.0–5.0)	0.172	7.0 (6.4–7.0)	0.001 **
Four years later	5.0 (5.0–5.0)	0.963	7.0 (6.0–7.0)	0.007 **
Five years later	5.0 (5.0–5.0)	0.852	7.0 (6.0–7.0)	0.001 **
Six years later	5.0 (5.0–5.0)	0.480	7.0 (6.0–7.0)	0.042 *

Data are presented as median (interquartile range). Wilcoxon signed-rank test was used for comparison. Statistical significance is indicated by * *p* < 0.05, and ** *p* < 0.01. MELD, model for end-stage liver disease.

**Table 5:** Change in noninvasive fibrosis score.

	APRI	*p*-Value (vs. Pre-Treatment)	FIB-4	*p*-Value (vs. Pre-Treatment)
Pre-treatment	0.6 (0.3–1.2)		2.4 (1.5–4.5)	
Baseline	0.4 (0.3–0.7)	<0.001 ***	2.1 (1.4–3.7)	<0.001 ***
One year later	0.4 (0.3–0.6)	<0.001 ***	2.0 (1.4–3.2)	<0.001 ***
Two years later	0.4 (0.2–0.6)	<0.001 ***	1.9 (1.2–3.2)	<0.001 ***
Three years later	0.4 (0.2–0.7)	<0.001 ***	2.0 (1.4–3.4)	<0.001 ***
Four years later	0.4 (0.3–0.6)	<0.001 ***	2.0 (1.4–3.2)	<0.001 ***
Five years later	0.4 (0.3–0.7)	<0.001 ***	2.2 (1.4–3.6)	<0.001 ***
Six years later	0.6 (0.3–1.0)	0.003 **	2.3 (1.7–4.9)	0.003 **

Data are presented as median (interquartile range). Wilcoxon signed-rank test was used for comparison. Statistical significance is indicated by ** *p* < 0.01, and *** *p* < 0.001. APRI, AST to platelet ratio index; AST, aspartate aminotransferase; FIB-4, fibrosis-4 index.

**Table 6:** Univariate and multivariate analyses for the risk factors for HCC occurrence after achieving SVR.

Variable	Univariate Analysis		Multivariate Analysis	
	HR (95% CI)	*p*-Value	HR (95% CI)	*p*-Value
Age > 71 years	8.722 (2.980–25.524)	<0.001 ***	5.142 (1.619–16.331)	0.005 **
AFP > 3.85 ng/mL	3.994 (0.865–18.434)	0.076		
Pre-FIB-4 > 2.227	9.954 (1.306–75.839)	0.027 *		
APRI > 0.556	3.961 (1.248–12.570)	0.019 *		
FIB-4 > 2.123	5.061 (1.126–22.352)	0.034 *		
Cirrhosis	4.057 (1.278–12.874)	0.017 *	5.173 (1.119–23.915)	0.035 *
Without cirrhosis				
Age > 71 years FIB-4 > 1.9407	16.053 (1.637–157.413) 77.312 (0.024–252,396.199)	0.017 ** 0.292	16.027 (1.635–157.119)	0.017 *

AFP, alpha-fetoprotein; APRI, AST to platelet ratio index; AST, aspartate aminotransferase; CI, confidence interval; FIB-4, fibrosis-4 index; HR, hazard ratio; HCC, hepatocellular carcinoma; SVR, sustained virologic response. Statistical significance is indicated by * *p* < 0.05, ** *p* < 0.01, and *** *p* < 0.001.

## Data Availability

Not applicable.

## References

[1] WHO (2021). WHO guidelines approved by the guidelines review committee. Recommendations and Guidance on Hepatitis C Virus Self Testing.

[2] Westbrook R.H., Dusheiko G. (2014). Natural history of hepatitis C. J. Hepatol..

[3] Lee S.S., Byoun Y.S., Jeong S.H., Kim Y.M., Gil H., Min B.Y., Seong M.H., Jang E.S., Kim J.W. (2012). Type and cause of liver disease in Korea: Single-center experience, 2005–2010. Clin. Mol. Hepatol..

[4] Chayama K., Takahashi S., Toyota J., Karino Y., Ikeda K., Ishikawa H., Watanabe H., McPhee F., Hughes E., Kumada H. (2012). Dual therapy with the nonstructural protein 5A inhibitor, daclatasvir, and the nonstructural protein 3 protease inhibitor, asunaprevir, in hepatitis C virus genotype 1b-infected null responders. Hepatology.

[5] Karino Y., Toyota J., Ikeda K., Suzuki F., Chayama K., Kawakami Y., Ishikawa H., Watanabe H., Hernandez D., Yu F. (2013). Characterization of virologic escape in hepatitis C virus genotype 1b patients treated with the direct-acting antivirals daclatasvir and asunaprevir. J. Hepatol..

[6] Hayashi K., Ishigami M., Ishizu Y., Kuzuya T., Honda T., Kawashima H., Ishikawa T., Tachi Y., Hattori M., Katano Y. (2018). Comparison of direct sequencing and Invader assay for Y93H mutation and response to interferon-free therapy in hepatitis C virus genotype 1b. J. Gastroenterol. Hepatol..

[7] van der Meer A.J., Wedemeyer H., Feld J.J., Hansen B.E., Manns M.P., Zeuzem S., Janssen H.L. (2014). Is there sufficient evidence to recommend antiviral therapy in hepatitis C?. J. Hepatol..

[8] Ioannou G.N., Feld J.J. (2019). What are the benefits of a sustained virologic response to direct-acting antiviral therapy for hepatitis C virus infection?. Gastroenterology.

[9] Terrault N.A., Hassanein T.I. (2016). Management of the patient with SVR. J. Hepatol..

[10] Singal A.K., Singh A., Jaganmohan S., Guturu P., Mummadi R., Kuo Y.F., Sood G.K. (2010). Antiviral therapy reduces risk of hepatocellular carcinoma in patients with hepatitis C virus-related cirrhosis. Clin. Gastroenterol. Hepatol..

[11] Kim N.J., Vutien P., Cleveland E., Cravero A., Ioannou G.N. (2023). Fibrosis Stage-specific Incidence of Hepatocellular Cancer After Hepatitis C Cure With Direct-acting Antivirals: A Systematic Review and Meta-analysis. Clin. Gastroenterol. Hepatol..

[12] Farhang Zangneh H., Wong W.W.L., Sander B., Bell C.M., Mumtaz K., Kowgier M., van der Meer A.J., Cleary S.P., Janssen H.L.A., Chan K.K.W. (2019). Cost Effectiveness of Hepatocellular Carcinoma Surveillance after a Sustained Virologic Response to Therapy in Patients with Hepatitis C Virus Infection and Advanced Fibrosis. Clin. Gastroenterol. Hepatol..

[13] Fujii H., Kimura H., Hasebe C., Akahane T., Satou T., Kusakabe A., Kojima Y., Kondo M., Marusawa H., Kobashi H. (2022). Real-world long-term analysis of daclatasvir plus asunaprevir in patients with hepatitis C virus infection. JGH Open.

[14] Nahon P., Bourcier V., Layese R., Audureau E., Cagnot C., Marcellin P., Guyader D., Fontaine H., Larrey D., De Lédinghen V. (2017). Eradication of hepatitis C virus infection in patients with cirrhosis reduces risk of liver and non-liver complications. Gastroenterology.

[15] Fujii H., Kimura H., Kurosaki M., Hasebe C., Akahane T., Yagisawa H., Kato K., Yoshida H., Itakura J., Sakita S. (2018). Efficacy of daclatasvir plus asunaprevir in patients with hepatitis C virus infection undergoing and not undergoing hemodialysis. Hepatol. Res..

[16] Suk K.T., Baik S.K., Yoon J.H., Cheong J.Y., Paik Y.H., Lee C.H., Kim Y.S., Lee J.W., Kim D.J., Cho S.W. (2012). Revision and update on clinical practice guideline for liver cirrhosis. Korean J. Hepatol..

[17] Sterling R.K., Lissen E., Clumeck N., Sola R., Correa M.C., Montaner J., Sulkowski M.S., Torriani F.J., Dieterich D.T., Thomas D.L. (2006). Development of a simple noninvasive index to predict significant fibrosis in patients with HIV/HCV coinfection. Hepatology.

[18] Wai C.T., Greenson J.K., Fontana R.J., Kalbfleisch J.D., Marrero J.A., Conjeevaram H.S., Lok A.S. (2003). A simple noninvasive index can predict both significant fibrosis and cirrhosis in patients with chronic hepatitis C. Hepatology.

[19] Lee H.W., Lee H., Kim B.K., Chang Y., Jang J.Y., Kim D.Y. (2022). Cost-effectiveness of chronic hepatitis C screening and treatment. Clin. Mol. Hepatol..

[20] Oh J.Y., Kim B.S., Lee C.H., Song J.E., Lee H.J., Park J.G., Hwang J.S., Chung W.J., Jang B.K., Kweon Y.O. (2019). Daclatasvir and asunaprevir combination therapy for patients with chronic hepatitis C virus genotype 1b infection in real world. Korean J. Intern. Med..

[21] Rockey D.C. (2019). Fibrosis reversal after hepatitis C virus elimination. Curr. Opin. Gastroenterol..

[22] Rosato V., Ascione A., Nevola R., Fracanzani A.L., Piai G., Messina V., Claar E., Coppola C., Fontanella L., Lombardi R. (2022). Factors affecting long-term changes of liver stiffness in direct-acting anti-hepatitis C virus therapy: A multicentre prospective study. J. Viral Hepat..

[23] D’Ambrosio R., Degasperi E., Anolli M.P., Fanetti I., Borghi M., Soffredini R., Iavarone M., Tosetti G., Perbellini R., Sangiovanni A. (2022). Incidence of liver- and non-liver-related outcomes in patients with HCV-cirrhosis after SVR. J. Hepatol..

[24] Cheetham T.C., Niu F., Chiang K., Yuan Y., Kalsekar A., Hechter R., Hay J.W., Nyberg L. (2015). Factors associated with failure to achieve SVR in hepatitis C Genotype 3 patients within an integrated care delivery system. J. Manag. Care Spec. Pharm..

[25] Nabulsi N.A., Martin M.T., Sharp L.K., Koren D.E., Teply R., Zuckerman A., Lee T.A. (2020). Predicting Treatment Failure for Initiators of hepatitis C virus Treatment in the era of Direct-Acting antiviral Therapy. Front Pharmacol..

[26] EASL (2018). EASL Clinical Practice Guidelines: Management of hepatocellular carcinoma. J. Hepatol..

[27] Marrero J.A., Kulik L.M., Sirlin C.B., Zhu A.X., Finn R.S., Abecassis M.M., Roberts L.R., Heimbach J.K. (2018). Diagnosis, Staging, and Management of Hepatocellular Carcinoma: 2018 Practice Guidance by the American Association for the Study of Liver Diseases. Hepatology.

[28] Kanda T., Lau G.K.K., Wei L., Moriyama M., Yu M.L., Chuang W.L., Ibrahim A., Lesmana C.R.A., Sollano J., Kumar M. (2019). APASL HCV guidelines of virus-eradicated patients by DAA on how to monitor HCC occurrence and HBV reactivation. Hepatol. Int..

[29] Korean Liver Cancer Association (KLCA) and National Cancer Center (NCC) Korea (2022). 2022 KLCA-NCC Korea practice guidelines for the management of hepatocellular carcinoma. Clin. Mol. Hepatol..

[30] Ghany M.G., Morgan T.R., AASLD-IDSA Hepatitis C Guidance Panel (2020). Hepatitis C guidance 2019 update: American Association for the Study of Liver Diseases-Infectious Diseases Society of America recommendations for testing, managing, and treating hepatitis C virus infection. Hepatology.

[31] EASL (2020). EASL recommendations on treatment of hepatitis C: Final update of the series (☆). J Hepatol..

